# “My world has shrunk”: a mixed-methods exploration of the impact of systemic autoimmune rheumatic diseases on patients’ lives

**DOI:** 10.1007/s00296-025-05993-2

**Published:** 2025-10-10

**Authors:** Martha A. Piper, Alice Tunks, Sean Humfrey, Lucy Calderwood, Shaista Tayabali, Sydnae Taylor, Arvind Kaul, Ellie Dalby, Shihab Ahmed, Sue Farrington, Thomas A. Pollak, Melanie Sloan

**Affiliations:** 1 Department of Public Health and Primary Care, University of Cambridge, Cambridge, UK; 2 The Wren Project, Pine Tree Nursery, Roundway, Devizes, UK; 3 London, UK; 4 Department of Rheumatology, St George’s Hospital, London, UK; 5 Institute of Psychiatry, Psychology and Neuroscience, King’s College London, South London and Maudsley NHS Foundation Trust, London, UK; 6 Faculty of Medicine and Health Sciences, University of East Anglia, Norwich, UK

**Keywords:** Systemic autoimmune rheumatic disease, Mixed methods, Impact, Relationships, Wellbeing, Identity, Surveys and questionnaires

## Abstract

Systemic autoimmune rheumatic diseases (SARDs) can involve physical, cognitive and mental health symptoms which have widespread impacts on patients’ lives. Research has found SARDs patients have lower quality of life and higher depression and anxiety compared to controls even after multivariate adjustment. Aim: This study aimed to explore how SARDs impact patients’ daily activities, work, relationships, wellbeing and identity. Methods: We used mixed-methods, collecting survey and interview data. In the survey, SARDs patients (*n* = 1853) completed the Warwick-Edinburgh Mental Wellbeing Scale (WEMWBS), our co-produced “ADAPT” measure, and questions about the frequency and impact of their symptoms. General population participants (*n* = 463) completed a modified survey. Survey responses were analysed using t-tests and correlation analyses. Interviews with SARDs patients (*n* = 34) explored the impact of SARDs on patients’ lives. Interviews and open-ended survey responses were analysed thematically. Results: Qualitative results highlighted how interacting, invisible and unpredictable symptoms impact patients’ relationships and participation in daily life. This was reflected quantitatively, where SARDs patients self-reported significantly lower satisfaction with life (mean difference=-13.244, *p* < 0.001) and participation in life (mean difference=-18.782, *p* < 0.001) compared to general population participants. Reduced participation, disbelief from others and changes in appearance damaged patients’ self-esteem and identity. In line with this, patients had significantly lower scores on every item of the WEMWBS compared to general population participants (overall mean difference=-6.467, *p* < 0.001). Physical, cognitive, mental, social and financial impacts of SARDs combined to adversely impact participation, relationships, and sense of self. Conclusion: Our findings call for the development of psychological, social and practical support options, in addition to improved disease control.

**Supplementary Information:**

The online version contains supplementary material available at 10.1007/s00296-025-05993-2.

## Introduction

Systemic autoimmune rheumatic diseases (SARDs) can have a far-reaching impact, with patients reporting moderate-to-severe impacts on their quality of life (QoL), work and daily activities, emotional well-being, personal relationships, and the lives of close relatives [1, 2]. SARDs patients have been found to have lower QoL and higher depression and anxiety scores compared to controls even after multivariate adjustment [3].

Health-related QoL (HRQoL) - the impact of health-related factors on an individual’s life - has been reported as significantly reduced in SARDs patients compared to healthy controls [4]. Cognitive impairment, defined as significant deficits in one or more cognitive domains including memory, attention and executive function, has been found to negatively impact both HRQoL and social role participation in SLE patients [5]. Poor HRQoL has also been linked to older age, fatigue and the presence of comorbid neurological or psychiatric disorders in SLE patients [6], and to physical and psychological symptoms in Sjögren’s patients [4]. SARDs can impact multiple systems such as the cardiovascular, pulmonary, renal, gastrointestinal, neurological and musculoskeletal systems, and this leads to greater reductions in QoL than if each system were affected in isolation [7].

Social support has been found to be associated with better health outcomes in SLE patients, including lower disease activity, less disease damage, and improved QoL [8]. Social worth and identity have also been reported to be damaged in SARDs patients due to inability to fulfil social roles [9]. Social media analysis of a rheumatoid arthritis (RA) forum identified that managing RA and its impact on relationships and social isolation negatively affects mental health, in particular feeling misunderstood by others [10]. Almost 90% of patients with autoimmune arthritis report feeling their illness is invisible, which is predictive of mental health difficulties [11].

As well as the direct effects of SARDs on the brain, several other factors have been found to contribute to the high prevalence of mental health symptoms in SARDs patients [12]. SARDs patients often experience uncertainty related to the unpredictable nature and severity of their symptoms, and this has been found to be positively correlated with depression, anxiety and psychological needs [13]. Physical limitations have also been found to predict depression levels, although this is bidirectional with levels of depression also predicting physical and psychological impairment [14]. Other factors such as the frequency of flares, recent hospitalizations and age of disease onset may increase the risk of developing mental health symptoms [11, 15].

This study aimed to provide an in-depth understanding of how living with SARDs impacts patients’ lives.

## Method

### Design

Quantitative data from the international INSPIRE survey included SARDs patients and general population participants (14). Qualitative data were collected via semi-structured interviews with SARDs patients and open-ended responses to survey questions about the impact of SARDs on patients’ lives.

### Patient and public involvement

Patients and clinicians were involved as equal members of our research team in every stage of this study, including developing the topic guide and survey, revising the themes and subthemes and presenting the findings.

### Materials

In the INSPIRE survey, SARDs patients completed the validated Warwick-Edinburgh Mental Wellbeing Scale (WEMWBS) and our newly developed, patient co-designed ADAPT measure which is currently undergoing validation in a randomised control trial [16, 17]. Patients were asked to self-assess their neuropsychiatric symptoms in terms of lifetime frequency from 0 (never) to 4 (always) and impact when experiencing each symptom from 0 (none) to 4 (“extremely negative impact, it makes my life very difficult when I have this symptom”). ADAPT measure items used for this study were: adapting to having a SARD, satisfaction with life, participation in life, and feeling of control. Items were scored from 0 to 100. A rigorous design and testing process, including using the “think aloud” method for checking understanding of survey questions with patients, ensured that questions were understandable and acceptable [18]. We followed recent guidelines on designing, conducting and reporting surveys [19].

The semi-structured interview schedule included guiding questions about how SARDs impacted patients’ lives. Interviewers were guided by each participant’s individual experiences and priorities, with appropriate probing of topics within the study’s aim. It was developed with the expertise of patient and clinician partners and informed by existing literature.

### Participants

Recruitment for the INSPIRE survey was conducted between 2022 and 2023 via social media, patient support groups and professional networks. SARDs patients were asked to forward a modified version of the survey to a friend without a SARD to form a comparison group [20]. The surveys were completed online in Qualtrics. Inclusion criteria were that all patients had to be 18 years or older and SARDs patients had to self-report a diagnosis of a SARD on clinical correspondence. General population participants self-reported that they did not have a SARD diagnosis. Patients were given a comprehensive list of SARDs at the start of the survey to select as appropriate, with the option of ‘other’ where patients could add a free text response. The free text responses were later assessed to see if they met SARD criteria. Patients who had opted in to being contacted for interview from the INSPIRE and related research group studies were purposively sampled to include a range of demographics and diseases.

### Procedure

Ethical approval was obtained through the Cambridge Psychology Ethics Committee on 8/7/2022: PRE 2022.027. All participants were given the participant information sheet and provided informed consent in Qualtrics before completing the survey and verbally before interviews. All data was kept in accordance with the General Data Protection Regulation [21].

### Analysis

Although survey data were collected before interviews were conducted, researchers only accessed it after interviews were complete, so neither analysis informed the other. The quantitative and qualitative findings were subsequently integrated according to “the triangulation protocol” [22]. This included consideration of areas of convergence and divergence between the two data sources, which were analysed and discussed by multiple co-authors including the lead and senior authors and patient co-investigators.

#### Quantitative

T-tests and bi-variate correlation analyses were performed in SPSS to investigate associations between variables of interest according to a pre-agreed statistical analysis plan. Correlations were calculated using Pearson’s coefficient and statistical significance was *p* = 0.05.

#### Qualitative

Based on the model of informational power, we deemed that a sample size of 34 interviews was sufficient given the broad research question, heterogeneity in participants’ disease type, the depth of the interviews, the additional 998 open-ended survey responses and thematic analysis [23]. Interviews were recorded and transcribed verbatim. The average duration of interviews was 58 min. Transcripts and survey responses were imported into NVivo and analysed using thematic analysis [24]. This included immersion in the data, initial coding and development of preliminary themes by MAP. Themes were developed iteratively, and all members of the study team, including patients, were involved in the generation and refinement of themes. Quotes were selected to illustrate themes and subthemes, labelled with participant numbers starting “S” for survey responses and “I” for interview responses.

### Reflexivity

Interviewers reflected on how their own biases, expectations and experiences influenced their interpretation of the data. All interviewers (MAP, AT, SAT, MS) had experience interviewing about sensitive topics including mental health and relationships. MAP and SAT had master’s degrees and AT and MS had PhDs. The participants had no relationship with the interviewers prior to the study. One interviewer had experience of living with a SARD and therefore had first-hand knowledge of the potential detrimental impacts on patient lives. The other interviewers were more naïve to the impact of SARDs and had a growing understanding of the topic, meaning they may have approached the interviews with fewer preconceptions but also potentially lacked the depth of insight to fully probe certain experiences. All interviewers remained reflective to ensure they were open to the non-negative impacts of SARDs. Interviewers had a range of sociodemographic characteristics including age and ethnicity, although all were female. Interviewer bias was managed by ensuring questions were clear and open-ended and by discussions with the multi-disciplinary team, including patient partners, throughout.

## Results

Of the *n* = 1853 SARDs patient survey participants, 93% were white and 91% were female (Table 1). The most common SARD was SLE, reported by 31% of survey respondents. General population respondents (*n* = 463) were also predominately white (94%) and female (72%). Open-ended responses were provided by 54% of survey respondents and approximately 55% of people invited for interview accepted and attended the interview. Non-attendance was mainly due to participants not responding to the email inviting them to interview, but a minority replied saying they were too busy or too unwell to participate in an interview.

**Table 1:** Participant characteristics

Characteristic	Interviews - SARDs patients (*n* = 34)		Survey - SARDs patients (*n* = 1853)		Survey - general population (*n* = 463)	
	Count	Percentage	Count	Percentage	Count	Percentage
Age						
18–29	4	12%	94	5.1%	45	9.7%
30–39	6	18%	195	10.5%	71	15.3%
40–49	8	24%	298	16.1%	82	17.7%
50–59	6	18%	519	28.0%	84	18.1%
60–69	2	6%	478	25.8%	120	25.9%
70+	7	21%	267	14.4%	60	13.0%
Prefer not to say	1	3%	2	0.1%	1	0.2%
Gender						
Female	28	82%	1687	91.0%	334	72.1%
Male	6	18%	160	8.6%	126	27.2%
Non-binary	0	0%	3	0.2%	3	0.6%
Other/undisclosed	0	0%	3	0.2%	0	0.0%
Disease						
Multiple primary	6	18%	145	7.8%		
Myositis	2	6%	64	3.5%		
Polymyalgia rheumatica (PMR)	2	6%	132	7.1%		
Rheumatoid/inflammatory arthritis (RA/IA)	2	6%	456	24.6%		
Sjögren’s	4	12%	150	8.1%		
SLE	11	32%	566	30.5%		
Systemic sclerosis (SSc)	3	9%	63	3.4%		
Vasculitis	2	6%	200	10.8%		
Undifferentiated connective tissue disease (UCTD)	2	6%	77	4.2%		
Ethnicity						
Asian	2	6%	49	2.6%	6	1.3%
White	26	76%	1718	92.7%	434	93.7%
Latino/a	3	9%	0	0.0%	0	0.0%
Black	2	6%	23	1.2%	4	0.9%
Mixed	1	3%	40	2.2%	11	2.4%
Other/undisclosed	0	0%	23	1.2%	8	1.7%

**Fig. 1:**
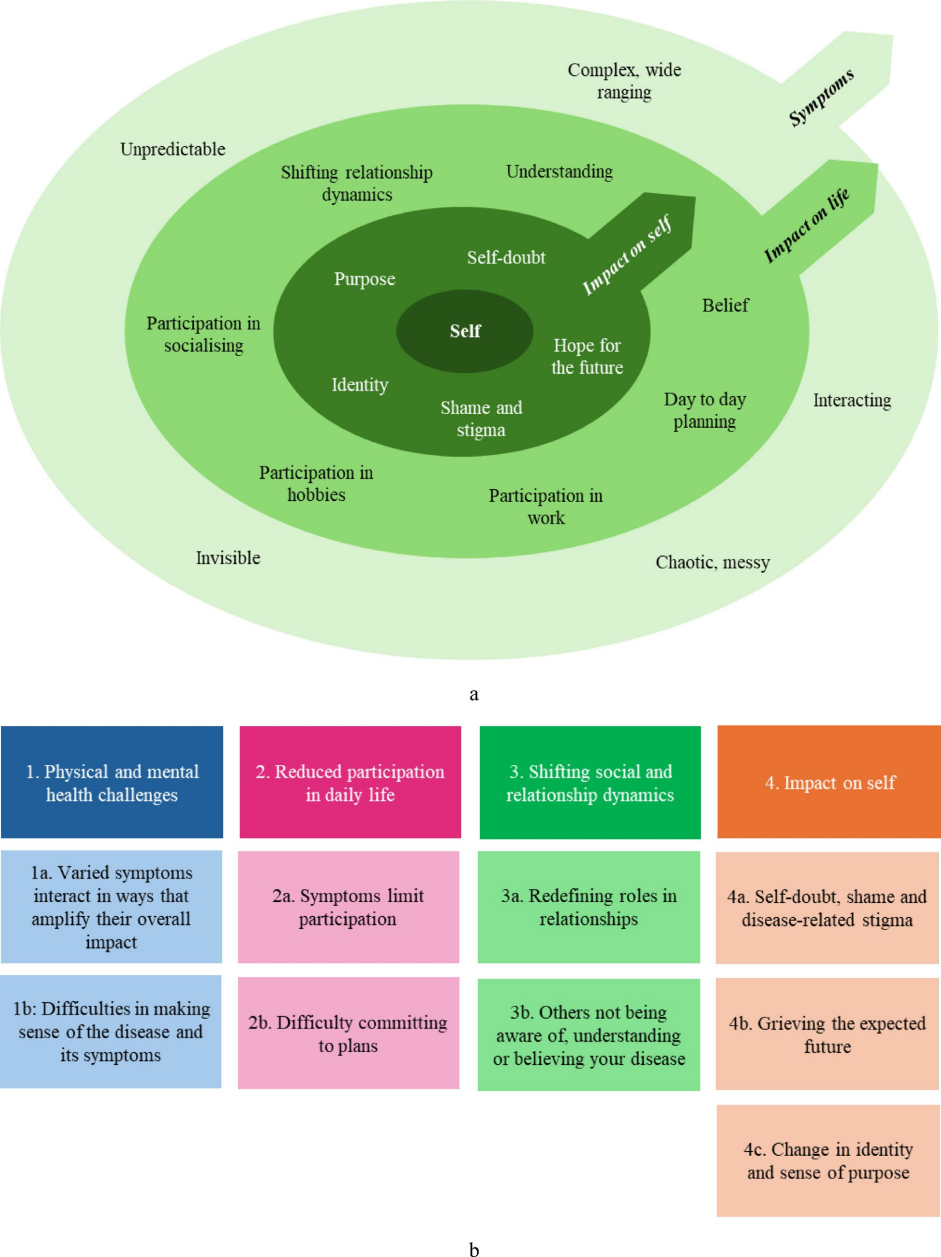
1**a** Diagram of impacts of disease on multiple domains of patients’ lives, 1**b** Diagram of themes and subthemes

Patients discussed losses in multiple domains of their lives, including abilities, energy, participation, relationships, independence, trust, confidence, identity, purpose and hope (Fig. 1a). Four themes were identified: (1) Physical and mental health challenges, (2) Reduced participation in daily life, (3) Shifting social and relationship dynamics, and (4) Impact on self. (Fig. 1b).

1. Physical and mental health challenges

1a. Varied symptoms interact in ways that amplify their overall impact: *“a vicious cycle of never feeling well”* (I18, UCTD, female, 30–39).

**Fig. 2:**
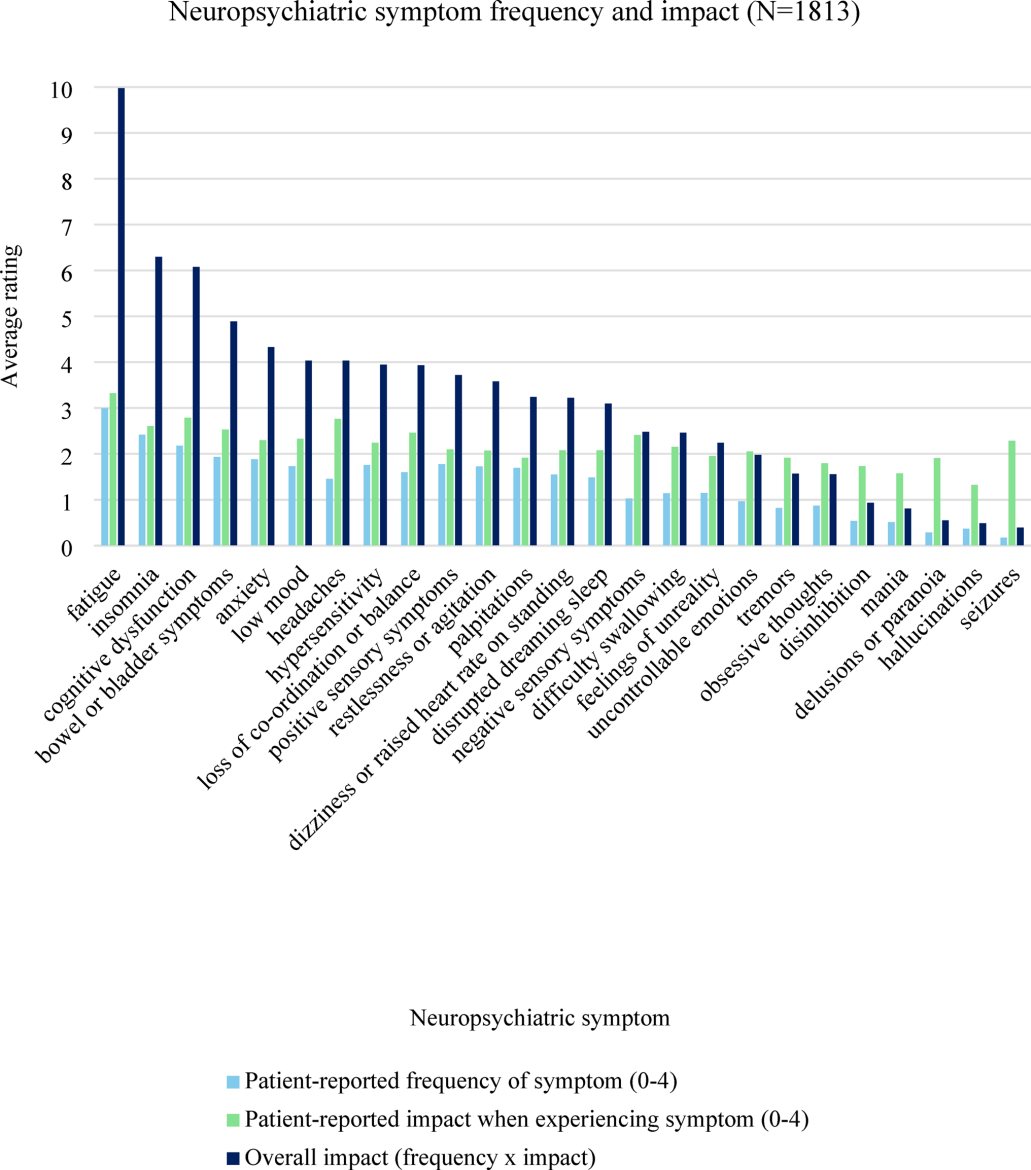
Neuropsychiatric symptom frequency and impact

Patients described a *“vicious cycle”* (multiple patients) of physical, cognitive and mental health symptoms. A wide range of symptoms were discussed, including systemic, dermatological, respiratory, ocular, musculoskeletal, urogenital, gastrointestinal, mental health, cognitive, neurological and sleep-related symptoms. Fatigue was rated as both the most impactful and most frequent neuropsychiatric SARD symptom, followed by insomnia, and cognitive dysfunction (Fig. 2).

The life-limiting effects of fatigue were also discussed in interviews.

*“I was getting tired and tireder, so tired I could just have curled up on a concrete pavement sort of thing”* (I06, SSc, female, 70+)

Cognitive symptoms included difficulties with memory, concentration and facial recognition. These symptoms made some patients feel “*embarrassed*” and “*old before my time”* (S1442, vasculitis, female, 60–69) and caused challenges communicating with others and performing daily activities.

*“I get to a certain point and it's like my brain just crashes and everything just goes blank, and a full reboot is required. Even simple things like constructing a sentence are difficult when my brain“crashes”. I will start a sentence and forget the other half of the sentence after about three words... It's very frustrating at times, as I am in my 20 s, the alleged prime of my life, and it makes me feel like a dementia sufferer.”* (S0505, vasculitis, female, 18–29)

Mental health symptoms were also discussed. These were considered by patients to be both a direct symptom of SARDs and a consequence of living with SARD symptoms, which limited their energy levels, QoL and ability to participate in life.

*“I feel my autoimmune disease is related to my mental health, I can feel a flare coming on as my mood lowers and anxiety rises”* (S1137, Sjögren’s, female, 50–59)

Many patients described experiencing “*constant pain*” (multiple patients), which affected their ability to engage in daily activities and impacted their mental health.

*“I am chronically mentally crippled which is closely coupled with physical pain. Every day is a war against pain, every day is a mental fight to behave with a degree of ‘normality’.” *(S0657, UCTD, female, 60–69)

Physical, cognitive and mental health symptoms were often described as simultaneous and mutually reinforcing; for example, pain and stiffness disrupted sleep, which increased cognitive difficulties, leading to anxiety, further disrupting sleep and increasing physical and mental fatigue.

*“I believe being constantly in pain, sick, and tired just increase physical and mental fatigue and vice versa. It's a vicious cycle of never feeling fully charged and well.”* (I18, UCTD, female, 30–39)

1b. Difficulties in making sense of the disease and its symptoms: *“it’s a massive jumble and trying to understand it is really hard”* (I21, SLE, female, age not disclosed).

Multiple factors such as co-diagnoses, treatments, hormonal changes and personal circumstances interacted with, masked and compounded the impact of SARDs. For patients with other health conditions, the similarity of symptoms made it difficult to distinguish their effects from those of the SARD(s).

*“The overlap in symptoms of fibromyalgia and Sjögren’s makes it difficult to identify which causes what.”* (S0671, Sjögren’s, female, 60–69)

Difficulty attributing symptoms to their SARD led to uncertainty and often made it difficult for patients to make sense of their disease.

*“That is lupus in a nutshell. Everything is like you can't pinpoint any of it. It's sort of like a massive jumble and trying to sort of understand it is really hard.”* (I21, SLE, female, age not disclosed)

Some patients also described how their disease had become an integral, normalised part of their life, which made it difficult to identify specific impacts.

Normalisation of symptoms appeared to occur particularly when the SARD had been present for a long time, as *“when you’ve lived with it so long and it’s like the normal*,* it’s quite hard to know how my life would have been different without it”* (I17, vasculitis, female, 50–59), and from childhood: *“I’ve never had that in inverted commas normality that other kids had.”* (I22, myositis, male, 40–49).

2. Reduced participation in daily life

**Fig. 3:**
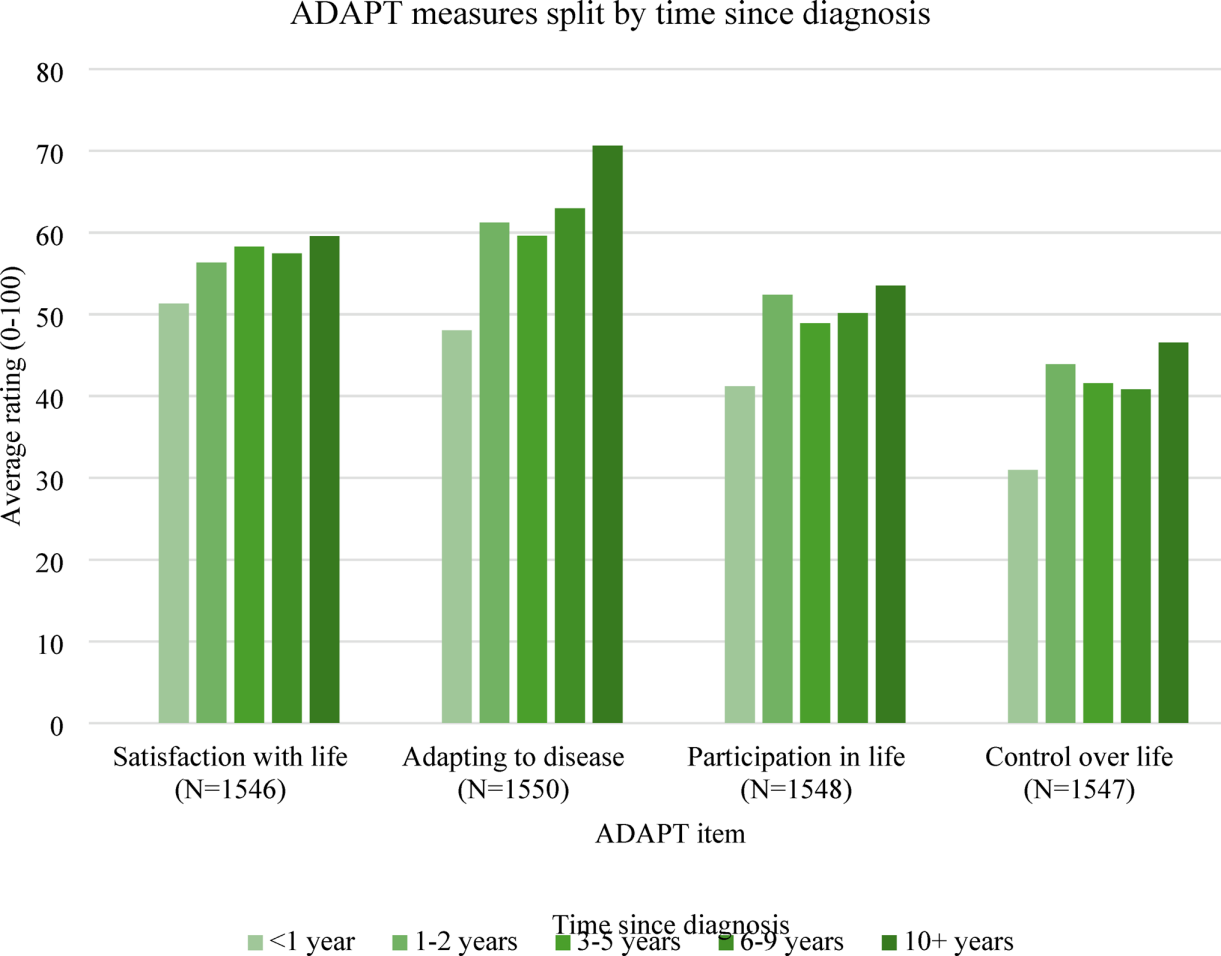
ADAPT measures split by time since diagnosis

There were no significant correlations between time since diagnosis and scores on the ADAPT items (Fig. 3). However, patients who had been diagnosed > 2 years ago had significantly higher scores compared to those diagnosed < 2 years ago on satisfaction with life (mean difference=−4.092, 95% CI [−7.094, −1.090], *p* = 0.008), adapting to disease (mean difference=−9.027, 95% CI [−11.911, −6.142], *p* < 0.001) and control over life (mean difference=−4.318, 95% CI [−7.652, −0.984] *p* = 0.011). Those diagnosed > 2 years ago also had higher scores for participation in life compared to those diagnosed < 2 years ago, but this difference was not significant. The greater challenges of adapting to and living with their disease when patients were younger and/or initially diagnosed were reflected in the qualitative data.

*“When you’re first diagnosed there is a lot to take in and no one seems to help you understand it or give you support” *(S0802, SLE, female, 30–39)

“*Now retired, I am able to pace myself and able to achieve most of what I need to do without worrying.” *(S0682, UCTD, female, 70+)

2a. Symptoms limit participation: *“my world has shrunk”* (multiple patients)

The complex, unpredictable and invisible symptoms of their diseases meant that many patients had had to withdraw from hobbies, social activities and work, which made them feel like *“my world has shrunk”* (multiple patients). The physical manifestations of their disease, such as musculoskeletal pain, mobility issues and fatigue, limited patients’ ability to engage in activities they enjoyed, which reduced quality of life.

*“My wife and I used to do a lot of hill walking and stuff like that. Totally out the question now. I used to do a lot of DIY. I still do a little bit, but not very much, and we used to go on quite a few holidays. That's all gone as well.”* (I14, RA/IA, male, 70+)

SARD symptoms often made it difficult for patients to work, with cognitive and physical challenges being referenced. Some patients had reduced working hours or stopped working altogether due to their SARD, either by choice or by medical necessity.

*“I got medically retired because of my illness and also because of my mental problems, I was getting all of my words mixed up and jumbled up.”* (I23, SLE, male, 70+)

Stopping work often led to financial difficulties and some patients were not able to claim benefits related to their SARD due to not meeting the eligibility criteria.

*“I am too poorly to work. I cannot contribute to the household finances. But I am not sick enough to claim our chronic illness cover on the mortgage. I am not well enough to hold a job down” *(S1097, SLE, female, 40–49)

Consequently, some patients had no choice but to work, which meant they were left with no energy to participate in activities outside of work.

*"I have to prioritise work in order to pay bills, but it means I have nothing left in me to do fun/social/nice things and if I want to have a night out, I have to book annual leave for the days surrounding the night/day so I can rest before and after. That's the hardest thing; it's made my life a lot smaller than it used to be.”* (S0689, SLE, female, 40–49)

Patients described withdrawing from socialising, with reasons including strict dietary requirements, fatigue and being immunosuppressed. They explained how they *“didn’t have as much energy as some of my friends”* (I04, multiple primary, female, 30–39) which made it difficult to participate in social activities.

*“My diet is extremely restricted. I fear eating out and being away from home in case of accidents, so my life has become limited”* (I24, SSc, female, 70+)

*“I am immune suppressed due to treatment and don't go out much or mix with many people. My world has shrunk, and I look inward a lot.”* (S0322, vasculitis, female, 50–59)

Reduced participation in hobbies, work and social activities meant that many patients lost contact with friends and found it difficult to meet new friends or romantic partners.

*“The peripheral ones, the ones who aren't really close friends, they forget about you naturally because you're not there.”* (I03, PMR, female, 60–69)

Sometimes patients had not realised the full impact of their disease until they looked back retrospectively to what their life was like pre-diagnosis.

*“Over the years you forget how much the condition has impacted your life as you adapt by slowing down [or] reducing or stopping activities altogether. It's only when you look back at what you used to be capable of that you realise the impact. It's quite depressing and I have a huge sense of not ‘living'or enjoying life, just drifting day to day.” *(S1522, myositis, female, 40–49)

2b. Difficulty committing to plans: *“better to just avoid*,* than to disappoint”* (S1195, SLE, female, 40–49)

Many patients described their symptoms as unpredictable. This was *“undermining”* (S1791, vasculitis, female, 50–59) and *“frustrating”* (I16, RA/IA, female, 18–29) as it hindered patients’ ability to plan. The unpredictability of symptoms also brought *“stress and anxiety in not knowing what is going to happen with your body hour to hour and day to day.”* (S0938, multiple primary, female, 50–59).

*“The worst aspect to me in this regard is not knowing how I will be on any given day, so although one can plan ahead, one's health may take a different view and ruin an event. I have no control over my body and do not like this!”* (S1166, PMR, female, 70+)

Patients expressed concerns about cancelling plans and letting people down. This deterred some patients from making any plans at all, deciding it was *“better to just avoid*,* than to disappoint”* (S1195, SLE, female, 40–49). Others explained that they felt obliged to go ahead with social plans even if it was detrimental to their health to avoid being excluded from social circles and future events. Some patients described how friends had stopped inviting them to social events, seeing them as “*unreliable*” (I09, Sjögren’s, female, 70+). This led to feelings of isolation and meant some patients struggled to re-integrate into friendships even when their symptoms remitted.

*“People have given up trying. Because they think, well, you know, she won't come out.” *(I17, vasculitis, female, 50–59)

Patients also discussed how changes to their appearance meant they were nervous about reuniting with friends or meeting new people. This sometimes led to self-ostracisation where patients only felt comfortable seeing a small number of family members or carers.

*“[In order to go back to work], I’d have to have a huge injection of confidence somehow, and a full set of gnashers.”* (I11, Sjögren’s, female, 60–69)

3. Shifting social and relationship dynamics

3a. Redefining roles in relationships: *“I feel like a burden”* (I16, RA/IA, female, 18–29).

Patients expressed frustration about how their SARD affected their relationship dynamics, as they relied more on others for support and those around them perceived and treated them differently.

*“I was a fiercely independent person but the symptoms, particularly fatigue, meant that I had to rely on others to do things for me, leaving me constantly frustrated and angry. I also realised that my family and friends began to treat me differently…, it made me feel even more disassociated from the post diagnosis version of me.”* (S1747, SLE, female, 60–69)

Many patients also expressed guilt about how their relationships had changed, as they were no longer able to do the things they used to do with their partner. They expressed feeling *“like a burden… like it’s a debt I can never repay”* (I16, RA/IA, female, 18–29).

*“I think it is irritating for my husband... I feel guilty that my husband doesn't have the lively, vital woman that he married. I feel very defeated sometimes.” *(S0434, PMR, female, 70+)

Concerns about the impact on children and grandchildren were discussed, with patients describing how their SARD had affected their children and wider family.

*“I worry that because of this disease that I'm not a good enough mum to my children.” *(S1185, RA/IA, female, 30–39)

**Fig. 4:**
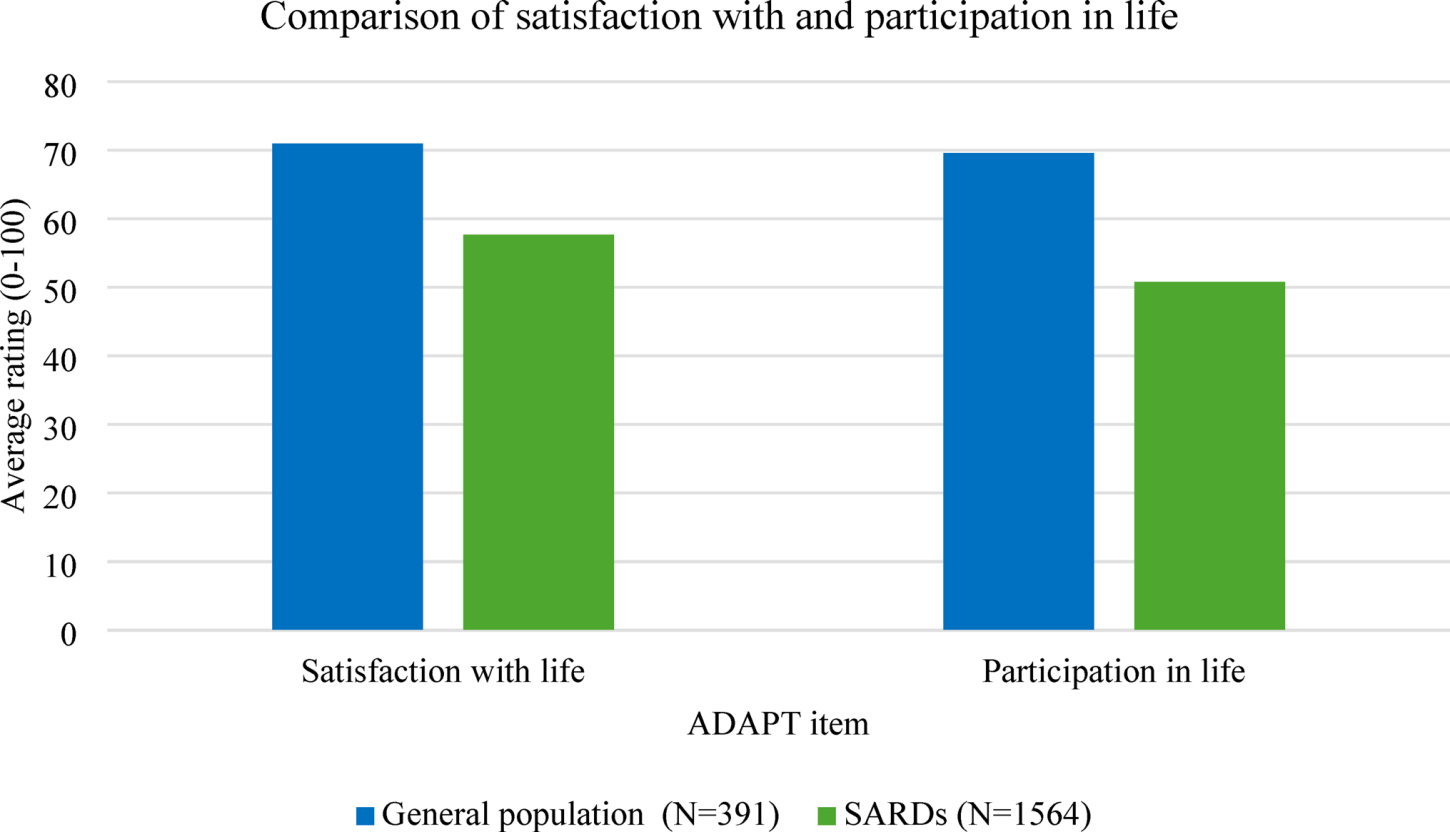
Comparison of satisfaction with life and participation in life

This was reflected in the quantitative data, where SARDs patients had significantly lower scores for satisfaction with life (mean difference=−13.244, 95% CI [−15.980, −10.508], *p* < 0.001) and participation in life (mean difference=−18.782, 95% CI [−21.832, −15.773] *p* < 0.001) compared to general population participants (Fig. 4).

3b. Others not being aware of, understanding or believing your disease: *“I look normal*,* so nobody understands how awful I feel inside”* (I07, Sjögren’s, female, 50–59).

Many patients explained how the “*invisible*” (multiple patients) nature of their symptoms affected the awareness of others because they looked unchanged from before the onset of their disease.

*“Because I look and behave like a perfectly normal healthy person and don't'look ill'people often expect too much of me, e.g. ask me to help them lift heavy things, etc, and then are disappointed with me when I can't. I feel like I'm swinging the lead sometimes.”* (S1613, RA/IA, female, 50–59)

Many patients also described SARDs as rare and not well-known, which contributed to the low levels of awareness among others. Low awareness, combined with the invisibility of their symptoms, meant that others could forget patients were unwell and expected more of them because they *“looked fine*” (multiple patients).

*“Before I got diagnosed, I'd started prepping the foundations to build a garden wall. And then I was being diagnosed, and my dad was just like you never did get the garden wall finished, why was that? And I'm like, I'm literally. I've literally just asked you to open a can of Diet coke for me because I can't open it. Like, what do you think's going on here?”* (I16, RA/IA, female, 18–29).

However, some patients felt that their friends understood and accepted their SARD and accommodated their needs.

*“Socially, it has not impacted much because I think I have people who kind of understand this and they don't really force me”* (I01, multiple primary, female, 18–29)

Often patients described how the invisibility of their disease meant that others didn’t believe that they were unwell and this had widespread consequences for patients’ relationships, self-esteem and in the workplace.

*“It has caused problems with work, as it is a hidden disease people do not understand that I need some help with certain things for example, aids for my computer, a chair for my neck and back. Having flare ups and not being able to move my hands easily. Being looked at as though there is nothing wrong with you.”* (S1560, RA/IA, female, 40–49)

4. Impact on self

**Fig. 5:**
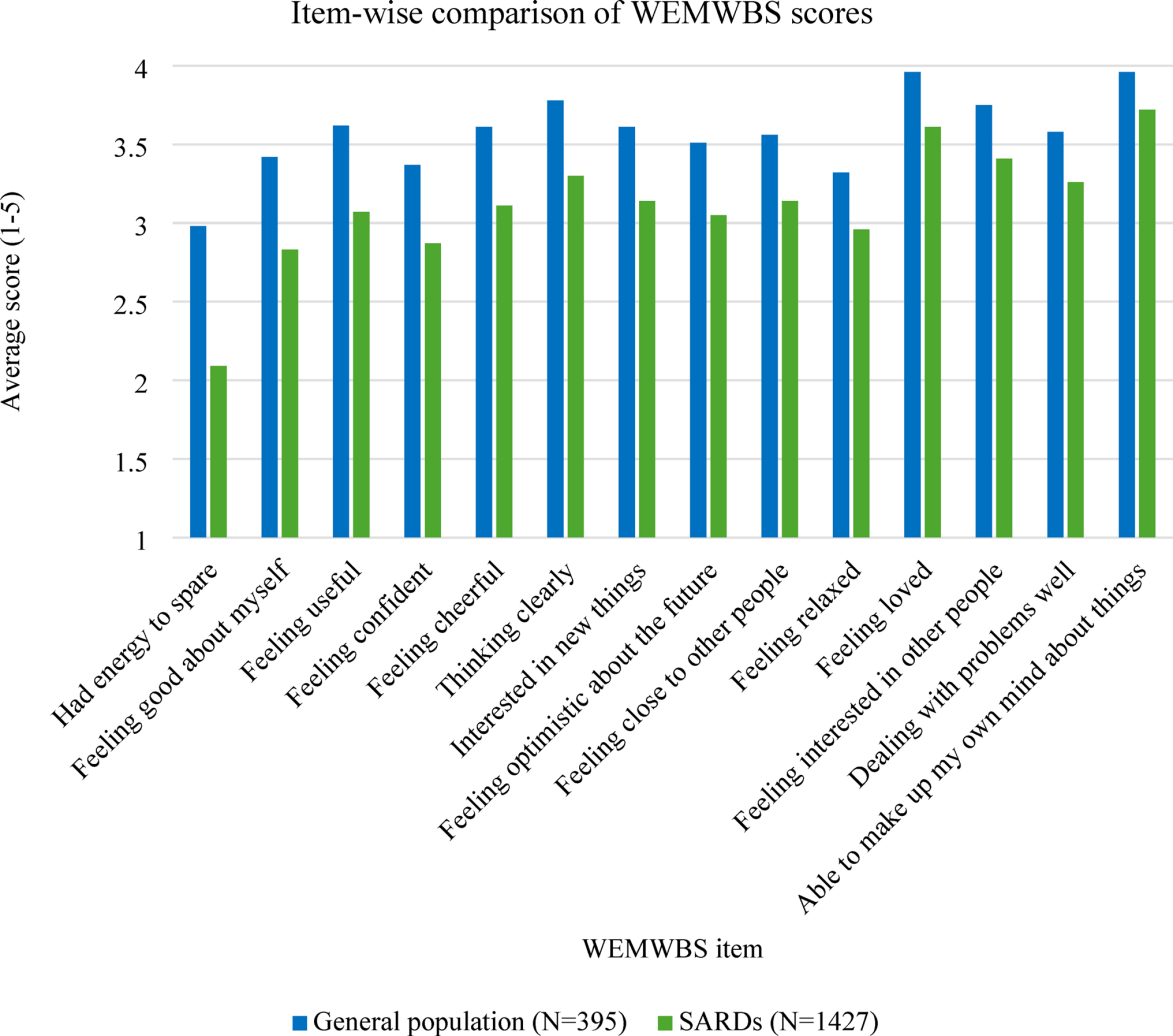
Item-wise comparison of WEMWBS scores

General population participants scored significantly higher than SARDs patients on the overall WEMWBS (SARD mean = 43.5606, general population mean = 50.0278, mean difference=−6.467, 95% CI [−7.640, −5.295] *p* < 0.001). General population participants also scored significantly higher than SARDs patients on every individual item of the WEMWBS (*p* < 0.001 for all items) (Fig. 5).

4a. Self-doubt, shame and disease-related stigma: *“disbelief has really damaged my self-esteem”* (S1791, vasculitis, female, 50–59)

Patients explained how not being understood or believed by loved ones, colleagues and doctors led to *“a lot of self-doubting”* (I34, SLE, female, 18–29). They recalled questioning whether their symptoms were so debilitating, or whether they were imagining them and *“using the conditions as an excuse”* (I12, multiple primary, female, 40–49).

Many patients expressed a sense of shame and embarrassment about being unwell with a chronic disease, especially if their symptoms were invisible to others. This was particularly the case for younger patients, as their symptoms necessitated a slower pace of life which differentiated them from others their age.

*“You almost feel a sense of shame that you're young and you've got something wrong with you... a certain shame that you were young, so you should be keeping up with everyone else.” *(I06, SSc, female, 70+)

*“The shame and embarrassment of using aids at 40 was horrendous to come to terms with and also the looks and stares of some people, judging a now 45 yr old using a mobility scooter.”* (S0672, RA/IA, female, 40–49)

Patients often reported that as they got older, living with a SARD became easier as *“everybody's slowing down at the level that I was slowing down.” *(I04, multiple primary, female, 30–39). This meant their disease didn’t set them apart from peers as much because common symptoms like fatigue, joint pain and brain fog were less stigmatised.

4b. Grieving the expected future: “I no longer feel able to dream” (S1624, SLE, female, 30–39)

Many patients explained that being diagnosed with a chronic disease had changed their long-term outlook and *“taken away my expected future”* (S0583, RA/IA, female, 60–69).

*“You become like a character in Little Women or something, like Beth. She wasn’t destined to grow up. She was destined to spend most of the novel on her back in bed. You became one of those characters as opposed to one of the ones that went off to somewhere and became a writer or something.”* (I28, SLE, female, 40–49)

Some patients described coping mechanisms to manage the uncertain and incurable nature of their disease, such as *“telling myself it’s temporary”* (I27, UCTD, female, 50–59). Others described how living with a chronic disease had *“made me much more resilient and patient”* (S0334, RA/IA, female, 40–49) as they navigated an unpredictable future and developed a more adaptive mindset.

4c. Change in identity and sense of purpose: *“I am half the person I was before I became unwell”* (S1963, SLE, female, 60–69).

Many patients’ identities had been tied to their roles in relationships, professions and hobbies, all of which were altered by the limitations of their disease. Many patients commented on losing their identity and *“grieving for the person I used to be before I was sick”* (I25, Sjögren’s, female, 50–59).

*“It has had a big impact because your identity is tied up with what you can do and how you look.”* (S0752, SSc, female, 40–49)

Several patients spoke about how their (former) profession had given them a sense of purpose, and how having to stop work made them feel *“worthless and unhappy”* (S1539, SLE, female, 50–59) and *“like I have failed professionally”* (S1877, Sjögren’s, female, 50–59). Some patients felt that their professional role was part of their identity, and so no longer being able to work in that role had disrupted how they saw themselves.

*“I don't feel I have a purpose anymore and I think that's one of the major things... This is an existence; it’s not a life. And just the social contact you get at work. You know, I miss it so much, and yes, it's horrible.”* (I11, Sjögren’s, female, 60–69)

Changes to patients’ identities, relationships, participation and future expectations was discussed as leading to a sense of *“bereavement for my old life”* (S1179, RA/IA, female, 50–59).

*“I am half the person I was before I became unwell, I don't recognise myself”* (S1963, SLE, female, 60–69)

## Discussion

This study presents qualitative and quantitative evidence of the wide-reaching, predominantly negative impacts of living with SARDs. The overwhelming impact is one of loss in multiple domains and reduced participation in many activities of daily life. The challenge of managing multiple interacting and unpredictable symptoms is compounded by the invisible nature of symptoms which can reduce the level of empathy and support from society, clinicians, employers, friends and family. Disbelief and misunderstanding, coupled with changes in appearance, identity, expected future and purpose, damaged patients’ self-esteem. Quantitative analysis of patient wellbeing scores demonstrated significantly lower overall wellbeing for patients than the general population on every individual item of the WEMWBS measure.

Qualitative and quantitative data were in agreement for many impacts. For example, patient wellbeing scores were significantly lower compared to the general population, particularly for items relating to energy, feeling good about yourself, feeling useful and feeling confident, all of which were prominent themes in interviews. There were some differences between the quantitative and qualitative results, such as insomnia being the second most commonly reported neuropsychiatric symptom but not discussed by many patients during interviews, where other neuropsychiatric symptoms such as depression and anxiety took precedence.

When asked about the impact of SARDs on their lives, patients often expressed initial bewilderment or frustration at the question and explained that it was impossible to define the impact because they did not know what their life would have been like if they hadn’t had their disease. SARDs were described as all-encompassing and life-altering, such that many patients could not imagine their lives without their disease. Patients described a cycle of physical, cognitive and mental symptoms, as individual symptoms could exacerbate or trigger additional symptoms, which is in keeping with existing literature [25, 26].

Both qualitative and quantitative data showed that disease symptoms prevented patients from participating in daily life as they had before disease onset. In common with other SARDs studies, fatigue was identified as the most life-changing and prevalent symptom and patients reported an impact on emotions, work and daily activities [27, 28]. This paper adds to the literature by describing how other symptoms such as cognitive and gastrointestinal symptoms also prevent patients from socialising as they used to. While many patients had lost most or all their friends due to not being able to work or socialise, some described how the challenges of their disease had reinforced and strengthened existing friendships and support networks. The impact on work is also reflected in the literature, with rates of unemployment found to be higher among RA patients compared to controls and increasing with disease duration and levels of disease activity [29]. Employment difficulties have also been identified among SLE patients, with barriers to work relating to the invisibility and unpredictability of symptoms [30]. Our study highlighted how inability to work affects patients across all SARDs and revealed additional challenges with eligibility for claiming associated benefits.

Patients reported how their reduced abilities had repercussions for relationships and social dynamics. This reflects existing studies where SSc patients reported needing caregiver support with completing daily tasks, and UCTD patients reported a change in their role and challenges upholding family responsibilities [31, 32]. Our study additionally found that increased reliance on support and a changed social role can lead to feelings of guilt and threaten patients’ self-esteem. This was supported by quantitative analysis showing that patients scored significantly lower on WEMWBS items “I’ve been feeling good about myself” and “I’ve been feeling confident” compared to general population participants.

A notable finding was that not being able to participate in work, socialising or hobbies during a period of increased disease activity seemed to reduce the likelihood of participating once symptoms remitted, as patients lost confidence in their abilities and lost contact with colleagues or friends. Many patients also described not being able to commit to social activities due to unpredictable disease symptoms. This is reflected in the literature which finds that the uncertainty of flare-ups leaves patients feeling helpless, physically limited and constantly fearing the next episode [33]. Some patients in our study described silver linings to living with an unpredictable disease, such as resilience, patience and sensitivity, and this has also been explored in previous studies [34]. Confidence in socialising was also impacted by changes in appearance, such as hair and teeth loss, and weight gain. This has been found to impact mental health, with body changes in SLE patients being associated with depression and anxiety, impacting QoL and leading to feelings of shame and a loss of identity [35].

SARDs were described by patients as poorly understood by others, which contributed to feelings of isolation and meant patients had to educate and manage the expectations of family, friends and work colleagues. Previous studies have found that SARDs have an amplified psychological impact on patients when not well-understood by family and friends [36]. In many cases, patients’ symptoms were invisible, and they reported not feeling believed, which further damaged their self-esteem and contributed to feelings of shame and stigma about having a SARD. Notably, younger patients expressed heightened shame over their perceived inability to meet expectations or keep up with peers, an experience less evident among older patients. Research suggests that internalised stigma about a patient’s own disease may contribute towards reduced self-esteem and make patients less likely to seek social support [37].

Lack of understanding and belief from those around them meant many patients reported little to no social support for their SARD. Our previous research has found that online peer support may reduce isolation and facilitate friendships between patients who can relate to one another’s experiences [9]. Other research highlights the importance of social support for reducing anxiety and depression and for promoting feelings of belonging and self-esteem, which are also associated with positive health outcomes [15, 38].

Changes to patients’ daily lives, appearance and relationships compounded one another, resulting in an upheaval of patients’ identity and sense of self. Hopes for the future often had to be re-evaluated within the framework of patients’ new identity and abilities, which impacted their mental health. This mirrors existing research which found that depression within SLE is linked to the challenges of feeling different to who one used to be, as well as coping with uncertainty [39]. Our quantitative data also reflected this, with SARDs patients having significantly lower scores on the WEMWBS item “I feel optimistic about the future” compared to controls.

Key considerations for policy and practice include increased awareness of the severe impacts of SARDs on multiple domains of patients’ lives, and the need to develop interventions to mitigate these identified impacts. Of particular importance is the need to design interventions that may reduce the observed withdrawal from many activities, and/or provide support for patients in finding alternative activities that can enhance their quality of life and self-esteem.

### Strengths and limitations

Strengths include the involvement of the multi-disciplinary team of researchers, patients and clinicians in all stages of the study. Another strength was the inclusion of multiple SARDs and impacts, which contrasts with many previous studies which focused on specific diseases or areas of life.

Despite purposive sampling, survey and interviewee demographics lacked diversity, with the majority of participants being White and female. There may have been selection bias in participants who attended interviews, as patients who were more unwell or impacted by their disease may not have been able to participate in interviews. Conversely, patients who had been more affected by their disease may have been more inclined to participate in interviews to have their experiences heard.

Furthermore, using patients’ friends as the control group may have introduced overmatching or selection bias if patients who were more socially connected or motivated were more likely to forward the survey on to a friend [40]. In addition, although most sociodemographic characteristics between patients and controls were similar, there was a higher proportion of males in the general population group, and we did not control for sociodemographic differences in our analysis. Self-reporting of SARD diagnosis also introduces the possibility that some participants did not have a formal diagnosis by a clinician. In addition, online recruitment methods may exclude those with limited technology skills or access. Attrition was observed throughout the survey, with 84% and 77% of SARDs patients completing the ADAPT and WEMWBS measures respectively, potentially introducing nonresponse bias [41].

Another limitation is that we did not control for age when comparing scores on ADAPT items between groups split by time since diagnosis (Fig. 3). Since groups with longer time since diagnosis may have an older average age, this may be a confounding variable.

Although this paper hasn’t explored the impact of medical relationships and treatment, we acknowledge the major role these also play in mental and physical symptoms and the consequent impact on daily life and patients’ sense of self [42].

## Conclusion

We found that SARDs impact patients’ lives in predominantly negative, wide-reaching and life-changing ways. Physical, cognitive, mental, social and financial impacts, among others, combine to adversely impact wellbeing, participation in daily life, relationships, and sense of self. Our findings highlight the unmet support needs of SARDs patients and call for the development of psychological, social and practical support options, as well as more effective medical treatment. Research into interventions which reduce the impact of SARDs on patients is vital to improve quality of life in this group.

## Supplementary Information

Below is the link to the electronic supplementary material.

Supplementary Material 1

## Data Availability

Anonymised data are available on reasonable request to the corresponding author.
